# Isolation of Myricitrin and 3,5-di-*O*-Methyl Gossypetin from *Syzygium samarangense* and Evaluation of their Involvement in Protecting Keratinocytes against Oxidative Stress via Activation of the Nrf-2 Pathway

**DOI:** 10.3390/molecules24091839

**Published:** 2019-05-13

**Authors:** Mansour Sobeh, Ganna Petruk, Samir Osman, Mohamed A. El Raey, Paola Imbimbo, Daria Maria Monti, Michael Wink

**Affiliations:** 1 Institute of Pharmacy and Molecular Biotechnology, Heidelberg University, 69120 Heidelberg, Germany; 2 AgroBioSciences Research Division, Mohammed VI Polytechnic University, Lot 660–Hay Moulay Rachid, Ben-Guerir 43150, Morocco; 3 Department of Chemical Sciences, University of Naples Federico II, Complesso Universitario Monte Sant’Angelo, via Cinthia 4, 80126 Naples, Italy; ganna.petruk@unina.it (G.P.); Paola.Imbimbo@unina.it (P.I.); 4 Department of Pharmacognosy, Faculty of Pharmacy, October 6 University, Giza 12585, Egypt; samirothman1965@yahoo.co.uk; 5 Department of Phytochemistry and Plant Systematics, National Research Center, Dokki, Cairo 12622, Egypt; elraiy@gmail.com

**Keywords:** *Syzygium samarangense*, antioxidants, myricitrin, 3,5-di-*O*-methyl gossypetin, eukaryotic cells, sodium arsenite

## Abstract

The wax apple (*Syzygium samarangense*) is traditionally employed as an antibacterial and immunostimulant drug in traditional medicine. This plant is rich in different flavonoids and tannins. In this study, we isolated two compounds from *S. samarangense* leaves: myricitrin and 3,5-di-*O*-methyl gossypetin. Then, we investigated the mechanisms of action of the two compounds against oxidative stress (induced by sodium arsenite) and inflammation (induced by UV light) on human keratinocytes. We could clearly demonstrate that the pre-treatment of cells with both compounds was able to mitigate the negative effects induced by oxidative stress, as no alteration in reactive oxygen species (ROS) production, glutathione (GSH) level, or protein oxidation was observed. Additionally, both compounds were able to modulate mitogen-activated protein kinase (MAPK) signaling pathways to counteract oxidative stress activation. Finally, we showed that 3,5-di-*O*-methyl gossypetin exerted its antioxidant activity through the nuclear transcription factor-2 (Nrf-2) pathway, stimulating the expression of antioxidant proteins, such as HO-1 and Mn-SOD-3.

## 1. Introduction

In the last 30 years, several lines of evidence demonstrate that oxidants play a crucial role in the etiology of aging. Reactive oxygen species (ROS) are considered responsible for the insurgence of many pathologies, such as malignancies, diabetes, inflammation, hypertension, atherosclerosis, cardiovascular diseases, liver diseases, and several types of bacterial and viral infections [1,2]. ROS can oxidize the nuclear base guanosine to 8-oxoguanosine, which could lead to point mutations and, as a consequence, to genetically mediated health conditions (as mentioned before).

The respiratory burst, cytochrome P450, lipid metabolism, and mitochondria represent the main sites for the generation of ROS [2,3]; however, numerous environmental stimuli, such as hyperthermia, chemotherapy, ultraviolet radiation, and intense exercise are considered as external sources of ROS [3,4].

To neutralize the deleterious effects of ROS, most organisms including humans are endowed with an innate antioxidant defense system. The latter includes endogenous enzymes, alpha lipoic acid, glutathione, vitamins A, C and E, and minerals such as Se, Mn, Cu and Zn that help to maintain redox homeostasis. When the intracellular redox equilibrium is disrupted, the oxidative stress pathway is activated and additional exogenous antioxidants, such as natural ones in food, may help to inactivate ROS. Indeed, it is now well established that natural antioxidants of dietary origin, for instance flavonoids and other polyphenols, ascorbic acid, and carotenoids, scavenge ROS and can effectively slow ageing or delay the progression of age-related diseases [3,4].

Originating from the Greater Sunda Islands, Malay Peninsula, and Andaman and Nicobar Islands, the wax apple, *Syzygium samarangense,* has gained a reputation as an antibacterial and immunostimulant medicinal plant. We recently identified 92 compounds from the leaf extract, mainly flavonoids and condensed tannins [5]. The extract exhibited robust antioxidant activity in vitro on immortalized human keratinocytes (HaCaT cells), and in rats, in which a substantial hepatoprotective activity against CCl_4_-intoxication was observed [5]. Recent research described five new triterpenoids, sysamarins A-D and sysamarin E (oleanane triterpenoid), in the leaf extract from plants grown in China [6].

In this study, we isolated two flavonoids, namely myricitrin and 3,5-di-*O*-methyl gossypetin (7,8,3′,4′-tetrahydroxy-3,5-dimethoxyflavone), from *Syzygium samarangense* leaves. Then, we evaluated the antioxidant activity in vitro and in HaCaT cells stressed with sodium arsenite. Moreover, we analyzed their antioxidant and anti-inflammatory properties at the molecular level, including in the mitogen-activated protein kinase (MAPK) and nuclear transcription factor-2 (Nrf-2) pathways.

## 2. Results and Discussion

### 2.1. Compound Isolation

#### 2.1.1. Myricitrin (Myricetin-3-O-α-rhamnoside) (Compound **1**)

The pure yellow amorphous material of compound **1** exhibited chromatographic characteristics (a dark purple spot on paper chromatography (PC) under UV light, turning to reddish orange when fumed with ammonia vapor or sprayed with Naturstoff specific for flavonoids) and UV absorption maxima in MeOH at 252 and 360 nm, which were identical to those reported for flavonol-3-*O*-glycosides [7]. Compound **1** exhibited [M − H]^−^ at *m/z* = 463 (ESI), and a main daughter ion at *m/z* 317 which corresponded for myricetin aglycone, suggesting the structure to be myricetin rhamnoside.

**Figure 1:**
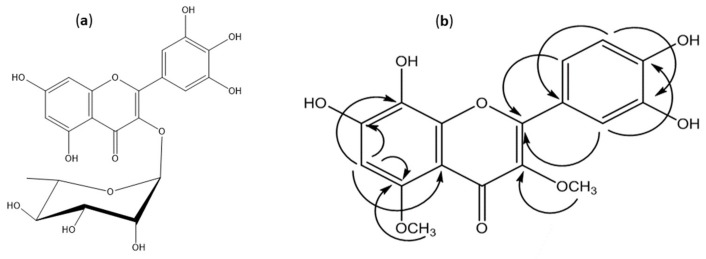
(**a**) Structure of compound **1**. (**b**) HMBC correlations of compound 7,8,3′,4′-tetrahydroxy-3,5-dimethoxyflavone (**2**).

^1^H-NMR spectroscopic analysis was carried out to confirm the structure. The spectrum (MeOD-d_4_) revealed, in the aromatic region, the characteristic pattern of myricetin proton resonances [8], and also revealed an anomeric proton resonance at δppm 5.23 (*J* = 1.5 Hz), assignable to the α- *L*-rhamnoside proton H-1′′. Final confirmation of compound **1** was achieved through ^13^C-NMR spectroscopic analysis in (MeOD-d_4_) and the data were consistent with those published before [9]. Therefore, the structure was assigned to myricitrin (myricetin-3-*O*-α-rhamnopyranoside, Figure 1) (^1^H- and ^13^C-NMR data, Table 1).

#### 2.1.2. 7,8,3′,4′-Tetrahydroxy-3,5-dimethoxyflavone (3,5-di-*O*-Methyl Gossypetin) (Compound **2**)

Compound (**2**) was obtained as a yellow amorphous powder. It showed a blue color under UV light (UV λmax: 256, 270, and 353). The NaOAc shift reagent test showed a 19 nm bathochromic shift (free 7-OH), suggesting that the structure of compound (**2**) is a flavonol substituted at positions 3 and 5 [8]. In negative ESI/MS, it showed a molecular ion [M − H]^−^ at *m/z* 345. ^1^H-NMR showed two methoxyl groups resonating at δppm 3.76 and 3.83 and one singlet at δppm 6.45, suggesting the hydroxylation of one A-ring proton. It was also observed that there was an absence of signals from 11 to 14 ppm, suggesting that OH-5 is occupied. In addition, characteristic proton patterns of 3,4-dihydroxy substituted B-ring [10]. For the precise determination of the site of hydroxylation and methoxylation, APT, HSQC, HMBC, and 2D NOESY analyses were performed.

In the HSQC spectrum of compound **2**, δppm 3.76 correlated to δppm 60.36 and in the HMBC spectrum correlated to 141.29 (C-3); this proved the attachment of the methoxy group to C-3. The other methoxy signal at δppm 3.83 will be then assigned to be 5-OCH_3_ due to the absence of a 5-OH signal. Also, 3.83 correlated to δppm 56.56 (HSQC), so it is not at 8-OH which resonates at about 60 ppm [11]. Resonance at δppm 3.83 is also correlated to 154.64 (HMBC) and correlated to δppm 6.45 (H-6) through the 2D-NOESY experiment. Therefore, OCH_3_ group (δppm 3.83) was assigned to be at C-5.

The APT experiment showed a signal at 127.47 assigned to C8-OH (about 133, if C6-OH) [11], thus, the site of hydroxylation was proved to be at C-8. Also, from the HMBC spectrum, δppm 6.45 resonance showed cross-peaks with signals at δppm 108.79 (C-4a), 154.64 (C-5), 152.93 (C-7) and 127.47 (C-8), which strongly supports that the signal is at H-6, rather than at H-7 or H-8. The signal at δppm 6.45 showed a cross-peak with the resonance at δppm 97.24 (HSQC) which is consistent with data published before [12], Figure 1. Therefore, the structure was identified as 7,8,3′,4′-tetrahydroxy-3,5-dimethoxyflavone (3,5-di-*O*-methyl gossypetin) (^1^H- and ^13^C-NMR data, Table 1).

### 2.2. In Vitro Antioxidant Activity

We first evaluated the in vitro antioxidant activity of the two isolated compounds using DPPH (2,2-diphenyl-1-picryl-hydrazyl-hydrate) and FRAP (radical scavenging and ferric reducing antioxidant power) assays. Both compounds exhibited substantial activities, as reported in Table 2. Similar activities were obtained from the total extract and positive controls [5].

### 2.3. Antioxidant Activity in a Cell-Based Model

#### 2.3.1. Biocompatibility in Human Keratinocytes

**Figure 2:**
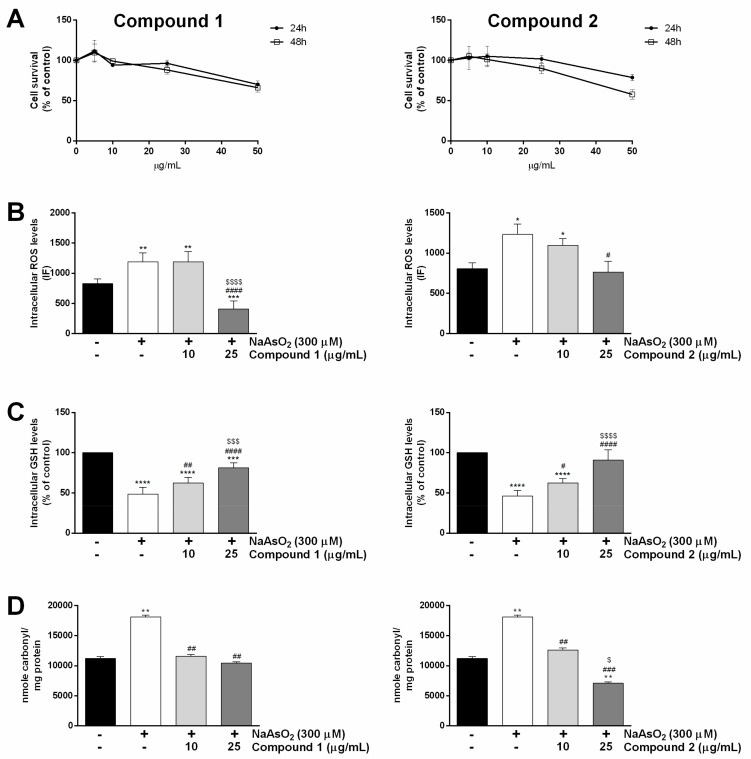
Effect of compounds **1** and **2** on human keratinocyte (HaCaT) cells. **A**: Dose–response curves of HaCaT cells after 24 h (black circles) and 48 h (empty squares) incubation in the presence of different concentrations of each compound. Cell survival rate was defined as in the Materials and Methods section. **B**–**D**: cells were pre-incubated in the presence of pure compounds (10 and 25 µg/mL) for 2 h and then exposed to 300 µM NaAsO_2_ for 60 min. **B**: intracellular reactive oxygen species (ROS) levels; **C**: intracellular glutathione (GSH) levels; **D**: protein carbonylation levels. Black bars refer to untreated cells, white bars to NaAsO_2_-treated cells, light grey bars to 10 µg/mL-treated cells and dark grey bars to 25 µg/mL-treated cells. Results are expressed as the means ± S.D. of three independent experiments, each carried out in triplicate. * indicates *p* < 0.05, ** indicates *p* < 0.01, *** indicates *p* < 0.001, **** indicates *p* < 0.0001, with respect to control cells; ^#^ indicates *p* < 0.05, ^##^ indicates *p* <0.01, *^###^* indicates *p* < 0.001, ^####^ indicates *p* < 0.0001, with respect to NaAsO_2_-treated cells; ^$^ indicates *p* < 0.05; ^$$$^ indicates *p* < 0.001, ^$$$$^ indicates *p* < 0.0001 with respect to 10 µg/mL-treated cells.

To verify that the isolated compounds were as nontoxic as the total extract [5], HaCaT cells were exposed to increasing amounts of compounds **1** and **2** (from 5 to 50 µg/mL) for different lengths of time. At the end of incubation, the MTT (3-(4,5-dimethylthiazol-2-yl)-2,5-diphenyltetrazolium bromide) assay was performed and the results are reported in Figure 2A. We found that cell viability was not affected by compounds **1** and **2** up to 25 µg/mL, independent of the length of the incubation (24 or 48 h). However, at a higher concentration (50 µg/mL), both compounds became cytotoxic for keratinocytes, more evidently in the case of compound **2** after 48 h of incubation. This finding is in line with our previous result obtained for the whole extract [5]. Indeed, we found that the extract from *S. samarangense* was cytotoxic when tested at concentrations higher than 100 μg/mL (after 48 h of incubation).

#### 2.3.2. Antioxidative Effect of Compounds 1 and 2 in HaCaT Cells

To investigate whether the pure compounds were able to prevent ROS generation in stressed keratinocytes, the fluorescent probe 2′,7′-Dichlorofluorescin diacetate (H_2_-DCFDA) was used. Sodium arsenite (NaAsO_2_) was used as a stress agent. Trivalent arsenic is one of the major contaminants of drinking water and air [13]. It acts as a prooxidant as it binds to critical thiol groups, which can inhibit important biochemical events thus leading to toxicity [14]. As shown in Figure 2B, ROS levels were significantly increased in cells after exposure to 300 μM NaAsO_2_ for 1 h (Figure 2B, white bars), whereas, when cells were pre-treated with 25 µg/mL of each pure compound, no significant alteration in ROS production was observed in both cases (Figure 2B, dark grey bars), with compound **1** being the most active. No protective effect was observed at 10 μg/mL (Figure 2B, light grey bars).

Subsequently, we analyzed the ability of the pure compounds to protect cells from NaAsO_2_-induced glutathione (GSH) depletion. GSH depletion is a direct consequence of ROS increase. We found that NaAsO_2_ caused a 50% GSH reduction (Figure 2C, white bars). Interestingly, when cells were pre-treated with pure compounds and then stressed, a dose-dependent inhibition of GSH depletion was observed in both cases (Figure 2C, light and dark grey bars). 

It is well documented that proteins are among ROS targets [15]. In particular, during protein oxidation, a stable carbonyl group is produced. Thus, carbonylation can be used as a marker of protein damage from prooxidants. Keratinocytes were treated as described above, and then carbonyl content was determined using 2,4-dinitrophenylhydrazine (DNPH). DNPH leads to the formation of stable dinitrophenyl (DNP) hydrazone adducts, which can be detected spectrophotometrically at 375 nm. The results obtained are reported in Figure 2D. As expected, exposure to NaAsO_2_ induced a high production of carbonyls (Figure 2D, white bars), whereas both compounds were able to maintain unaltered carbonyl levels already at 10 μg/mL. Interestingly, compound **2**, when tested at the highest concentration, significantly decreased carbonyl content with respect to untreated cells (Figure 2D, on the right, dark grey bar). These results agree with the data of Büchter et al. [16], who investigated the protective activities of the flavonoid myricetin in Hct116 human colon carcinoma cells against a lethal dose of H_2_O_2_.

**Figure 3:**
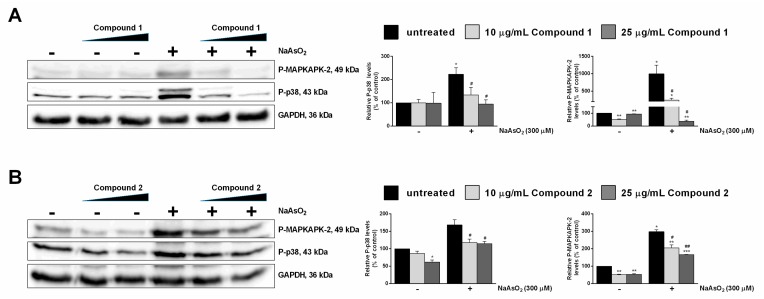
Effect of pure compounds on NaAsO_2_-induced mitogen-activated protein kinase (MAPK) cascade in HaCaT cells. Cells were pre-treated with compounds **1** (**A**) and **2** (**B**), as described in Materials and Methods, and Western blots were performed. The phosphorylation levels of MAPKAPK-2 (**A**, **B**, upper panel) and p38 (**A**, **B**, middle panel) are reported. Glyceraldehyde 3-phosphate dehydrogenase (GAPDH) was used as an internal standard. The relative densitometric analysis is reported. Black bars refer to control cells in the absence (−) or in the presence (+) of NaAsO_2_; light grey bars refer to 10 µg/mL-treated cells; and dark grey bars refer to 25 µg/mL-treated cells. Data shown are the means ± S.D. of three independent experiments. * indicates *p* < 0.05, ** indicates *p* < 0.01, *** indicates *p* < 0.001, with respect to control cells; ^#^ indicates *p* < 0.05, ^##^ indicates *p* < 0.01 with respect to NaAsO_2_-treated cells.

The protective effect exerted by both isolated compounds was further confirmed by analyzing the MAPK cascade pathway. The phosphorylation levels of p38 and of its direct target MAPKAPK-2 were analyzed in the presence of an increasing amount of each compound, and in the presence of oxidative stress [17]. As shown in Figure 3, both compounds protected human keratinocytes against NaAsO_2_, with compound **1** being the most active.

##### Compound 2 Stimulates Nrf-2 Nuclear Translocation and Nrf2-ARE Transcriptional Activity 

Flavonoids have been shown to exert their antioxidant activity by modulating one or more redox-sensitive transcription factors, such as nuclear factor (erythroid-derived 2)-like 2 (Nrf-2), fork-head box O (FoxOs) and/or peroxisome proliferator-activated receptor gamma (PPARγ) [18]. Therefore, in order to analyze the mechanism of action of compounds **1** and **2,** the role of the Nrf-2 pathway was evaluated. Nrf-2 is a leucine zipper transcription factor which, under physiological conditions, is negatively regulated by its repressor, Kelch-like ECH-associated protein 1 (Keap-1), which directs Nrf-2 to proteasomal degradation [19]. Upon stimulation by prooxidants or small amounts of antioxidants [19,20], Nrf-2 dissociates from Keap-1 and translocates into the nucleus where it binds to antioxidant response elements (AREs), with the consequent transcription of many genes coding for antioxidant proteins, such as heme oxygenase-1 (HO-1), superoxide dismutase (SOD), NAD(P)H quinone oxidoreductase 1 (NQO1), and γ-glutamate-cysteine ligase (γ-GCLC) [21,22]. Thus, nuclear Nrf-2 levels were analyzed by Western blot after 5 and 15 min incubation with 25 µg/mL of each compound, as these incubation times have been previously used to verify Nrf-2 nuclear translocation [23,24].

**Figure 4:**
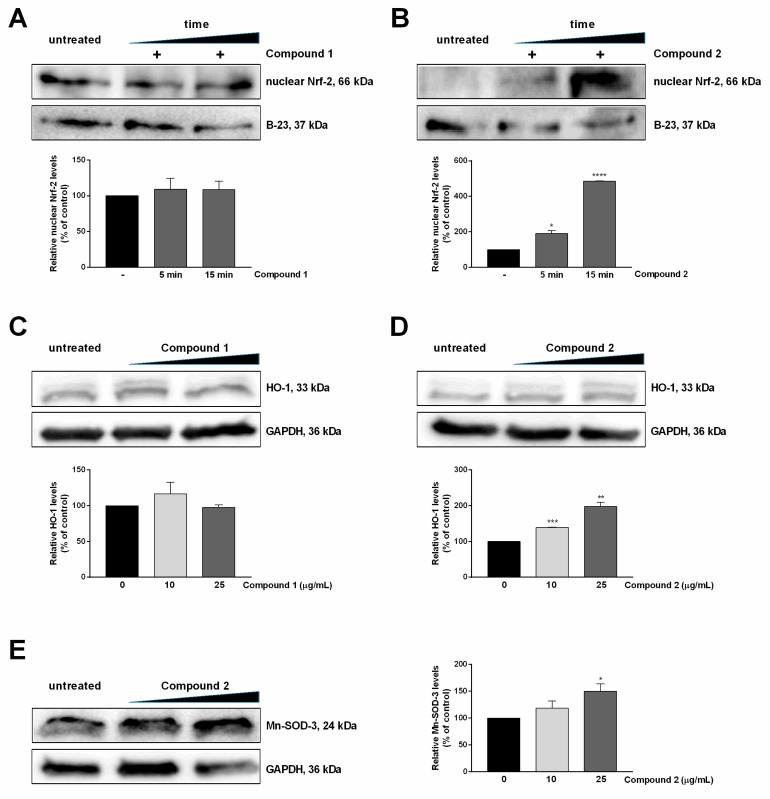
Mechanisms of action of isolated compounds. **A**,**B**: Nuclear transcription factor-2 (Nrf-2) levels were analyzed by Western blot. Keratinocytes were incubated with increasing concentrations of compound **1** (**A**) or compound **2** (**B**) for 5 min or 15 min and then lysed. The relative changes in Nrf-2 levels were quantified densitometrically; B-23 was used as a loading control. **C**,**D**: Western blot analysis for HO-1. **E**: Western blot analysis for Mn-SOD-3 of cells incubated with compound **1**. HO-1 and Mn-SOD-3 levels were quantified by densitometric analysis and normalized to GAPDH. Data shown are the means ± S.D. of three independent experiments. * indicates *p* < 0.05, ** indicates *p* < 0.01, *** indicates *p* < 0.001, **** indicates *p* < 0.0001, with respect to control cells.

As shown in Figure 4A, no effect of compound **1** was observed on the nuclear translocation of Nrf-2, whereas compound **2** (Figure 4B) clearly induced the nuclear translocation of this transcription factor. This result is very interesting for compound **2** as this is the first report on the mechanism of action for this molecule. Moreover, the failure of compound **1** to activate the Nrf-2 pathway is not so surprising, as there is controversy in the literature regarding the pathways activated by flavonols [25,26]. In particular, the most studied flavonol, i.e., quercetin, has been reported to activate Nrf-2 and/or the cytosolic aryl hydrocarbon receptor (AhR). Indeed, recent studies have indicated that Nrf-2 can be activated either directly by flavonols, or downstream activated after AhR activation [26,27]. Moreover, longer exposure (1–48 h) to flavonols may be required to witness Nrf-2 activation [27,28]. Thus, it is conceivable that in our experimental system, the incubation time used was not long enough to activate Nrf-2 nuclear translocation. The effective activation of AREs by compounds **1** and **2** was next confirmed by analyzing the intracellular levels of HO-1 (Figure 4C,D, respectively) and Mn-SOD (Figure 4E, only for compound **2**). As expected, compound **2** was able to activate the expression of phase II detoxifying enzymes, whereas compound **1** failed.

### 2.4. Pure Compounds as a Shield against UVA-Mediated Inflammation

**Figure 5:**
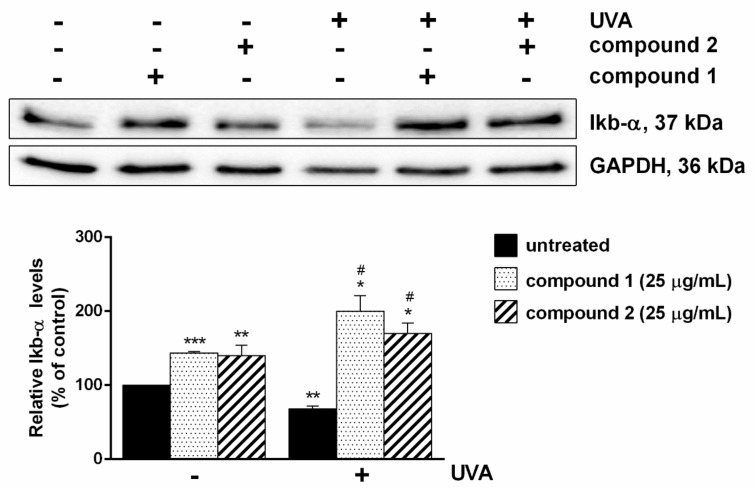
Effects of isolated compounds on inflammation. Cells were pre-treated with each compound (25 µg/mL) for 2 h. Then, cells were exposed to UVA (100 J/cm^2^) for 10 min and incubated at 37 °C for 30 min. Total lysates were resolved by 15% SDS-PAGE and the level of IκB-α was determined by Western blot analyses. All the values were normalized by using GAPDH as a loading control. The relative densitometric analysis, in the absence (black bars) or in the presence of each compound (dotted bars for compound **1** and diagonal lines bars for compound **2**) is reported. Data shown are the means ± S.D. of three independent experiments. * indicates *p* < 0.05, ** indicates *p* < 0.01, *** indicates *p* < 0.001, with respect to control cells; ^#^ indicates *p* < 0.05, with respect to UVA-treated cells. In the graph, (−), is referred to not irradiated cells, whereas (+) is refereed to cells irradiated by UVA.

Finally, the ability of the two compounds to inhibit inflammation was analyzed. To this purpose, inflammation was induced on HaCaT cells by UVA irradiation, as it has been reported that UVA is able to activate the inflammation pathway [29]. Cells were treated with UVA in the presence or in the absence of each compound. Western blotting analyses, using a specific antibody against IκB-α, were performed. IκB-α is one of the key regulatory elements which plays a central role in NF-κB regulation. Under physiological conditions, IκB-α acts as a physical inhibitor of NF-κB, whereas, upon inflammation stimuli, the two proteins dissociate, IκB-α is degraded and NF-κB translocates to the nucleus. As shown in Figure 5, a drastic decrease in IκB-α level was observed only after UVA treatment, whereas the pre-treatment of cells with the pure compounds was able to inhibit IκB-α degradation and consequent inflammation. These results are in agreement with the those reported in literature, in which different flavonols have been shown to inhibit inflammation [30].

## 3. Materials and Methods

### 3.1. Extraction and Isolation of Compounds

*S. samarangense* (Blume) Merr. & L. M. Perry (syn. *Eugenia javanica* L.) leaves were collected during the spring season in 2014. A plant sample is kept at the Pharmacognosy Department, Faculty of Pharmacy, Ain Shams University (Cairo, Eygpt) under PHG-P-SS-182 [31]. Air-dried leaves (300 g) were ground and extracted using methanol (3×1 L). The combined extracts were filtered and consequently evaporated under a vacuum at 40 °C until dryness, yielding a semisolid extract. The latter was then subjected to lyophilization, yielding 40 g. The lyophilized extract was then defatted with n-hexane and finally subjected to sequential fractionation using ethyl acetate, yielding 4 g, and butanol, yielding 3 g. 

A portion of the ethyl acetate fraction (900 mg) was applied to a medium-chromatography system (Puriflash 4100, Interchim, 03100 Montluçon, France) equipped with silica flash columns (PuriFlash column 30 Silica HP - 25.0 g, 22 bar). Elution was performed using a gradient of dichloromethane (A) and methanol (B). At 0 min, the ratio of B was 30% and reached 100% at 90 min. The flow rate was 15 mL/min and the UV detection was conducted at 254 and 365 nm. Three major fractions were obtained, which were further subjected to TLC screening, followed by purification on several Sephadex LH20 columns using methanol as eluent. Finally, two pure isolated compounds, namely myricetin-3-*O*-α-rhamnoside (myricitrin) (**1**) and 3,5-di-*O*-methyl gossypetin (7,8,3′,4′-tetrahydroxy-3,5-dimethoxyflavone) (**2**) were obtained.

### 3.2. In Vitro Antioxidant Activity

2,2-Diphenyl-1-picryl-hydrazyl-hydrate (DPPH) radical scavenging and ferric reducing antioxidant power (FRAP) assays were used to determine the antioxidant properties of the isolated compounds, and were carried out as described earlier [32].

### 3.3. Cell Culture 

The immortalized human skin keratinocyte cell line (HaCaT) was obtained from American Type Culture Collection (ATCC, Manassas, VA, USA) and was cultured in complete growth medium in a 5% CO_2_ humidified atmosphere at 37 °C, as described before [17]. Subculture was carried out in a ratio of 1:4–1:5 every 48–72 h.

The biocompatibility of the compounds towards the cells was assessed by the MTT (3-(4,5-dimethylthiazol-2-yl)-2,5-diphenyltetrazolium bromide) assay, as described before [17]. In brief, HaCaT cells were plated at a density of 2 × 10^4^ cells/cm^2^. The next day, the medium from each well was aspirated and 100 µL of fresh growth medium containing increasing amounts of pure compounds (from 5 to 50 µg/mL) was added and left for 24 and 48 h. Two groups of cells were used as a control, i.e., untreated cells and cells supplemented with identical volumes of methanol. The biocompatibility of each compound was expressed as the percentage of viable cells in the presence of the compound under testing, compared to the average of the control cells. Each sample was tested in three independent analyses, each carried out in triplicate.

### 3.4. Induction of Oxidative Stress with Sodium Arsenite

To evaluate the protective effect of the pure compounds against oxidative stress, HaCaT cells were pre-treated with 10 and 25 µg/mL of the respective compound for 2 h. At the end of incubation, cells were exposed to 300 µM sodium arsenite (NaAsO_2_) for 1 h. Immediately after oxidative stress induction, ROS production, GSH levels, protein carbonylation levels, and activation of mitogen-activated protein kinase (MAPK) cascade were determined by DCFDA assay, DTNB (5,5′-dithiobis-2-nitrobenzoic acid) assay, DNPH assay and Western blotting, respectively, as described below. 

### 3.5. DCFDA Assay

To examine whether the pure compounds may interfere with free radical propagation, the accumulation of intracellular ROS levels was measured by DCFDA assay. In brief, 24 h after seeding, 10 and 25 µg/mL of each compound (dissolved in Dulbecco’s Modified Eagle’s Medium (DMEM) without phenol red) were added to the culture medium. After 2 h of incubation, cells were incubated with 25 µM 2′,7′-dichlorodihydrofluorescein diacetate (H_2_-DCFDA, Sigma-Aldrich) for 45 min at 37 °C in complete medium without phenol red. At the end of incubation, cells were incubated with 300 µM NaAsO_2_ as described above. Cells were washed with warm PBS (phosphate buffer saline) supplemented with 1 mM CaCl_2_, 0.5 mM MgCl_2_, and 30 mM glucose (PBS plus) between each step. Then, the fluorescence intensity of the DCF probe was measured by using a Synergy™ HTX Multi-Mode Microplate Reader (excitation = 485 nm, emission = 535 nm, scanning speed = 300 nm/min and 5 slit widths for excitation and emission, BioTek Instruments, Inc., Winooski, VT, USA). ROS production was expressed as the DCF fluorescence intensity of the samples under testing. The results are given as the average of three independent experiments, each carried out in triplicate.

### 3.6. DTNB Assay

Total glutathione (GSH) content was determined by 5,5′-dithiobis-2-nitrobenzoic acid (DTNB) assay as described before [17]. The GSH content was expressed as the percentage of thio-2-nitrobenzoicacid (TNB) of the sample under testing, with respect to the untreated sample. The GSH content was extrapolated from absorbance values, obtained at 412 nm, based on the standard curve obtained by incubating the DTNB reagent with GSH (1–500 µM). The results are given as the average of three independent experiments, each carried out in triplicate.

### 3.7. DNPH Assay

To analyze protein carbonyl content, cells were treated as described above and then 2,4-dinitrophenylhydrazine (DNPH) assay was performed by using the protocol of the protein carbonyl content assay kit (Sigma-Aldrich, St Louis, MO, USA).

The carbonyl content was calculated using the equations below:

(1) $$\mathrm{Carbonyl}\;\mathrm{content}\;=\;\frac{\mathrm{nmolecarbonyl}}{\mathrm{mgprotein}}$$

(2) $$\mathrm{nmole}\;\mathrm{carbonyl}\;=\;\frac{Abs375nm}{6.364}\;\times \;100$$

where: 100 = total volume in well (µL); 6.364 = millimolar coefficient of extinction (ε^mM^ = 22 mM^–1^ cm^–1^) × 0.2893 cm path length in a well; Abs 375 nm = absorption at 375 nm of DNPH, which is related to carbonyl content. Three independent experiments were carried out, each one with three determinations.

### 3.8. Induction of Inflammation by UVA Light Treatment

To evaluate the ability of each compound to counteract inflammation, HaCaT cells were plated as described above. After seeding, cells were pre-treated with 25 µg/mL of the compounds for 2 h. At the end of incubation, the cells were washed with phosphate buffer saline (PBS) and then covered with a thin layer of PBS and exposed to UVA light (365 nm) for 10 min (100 J/cm^2^). Then, cells were washed twice with PBS and incubated in complete medium for 30 min at 37 °C. Inflammation was evaluated by Western blotting, as described below.

### 3.9. Western Blotting Analysis

To analyse MAPK cascade and inflammatory markers, cells were detached at the end of the treatment, and then lysed by re-suspending cell pellets in 50 μL of lysis buffer (300 mM NaCl, 0.5% NP40 in 100 mM Tris-HCl, at pH 7.4) supplemented with proteases and phosphatase inhibitors. To analyze nuclear transcription factor-2 (Nrf-2) activation, nuclear lysates were used. In the first step, cytosolic proteins were extracted by using PBS buffer containing 0.1% Triton and protease inhibitors. Then, the pellets, containing nuclear proteins, were resuspended in Radioimmunoprecipitation (RIPA) buffer (150 mM NaCl, 1% NP-40, 0.1% SDS, protease inhibitors in 50 mM Tris-HCl, at pH 8.0). For each condition, lysates (100 μg proteins) were analyzed by Western blotting performed as described before [33]. Specific antibodies against phosphorylated p38 and MAPKAPK-2, total Mn-SOD and Nrf-2 were purchased from Cell Signal Technology (Danvers, MA, USA). HO-1 antibody was from Bethyl (Montgomery, TX, USA). IκB-α antibody was from Santa Cruz Biotechnology, Inc. (Santa Cruz, CA, USA). To normalize protein intensity levels, a specific antibody was used for total (anti-GAPDH, Thermo-Fisher, Rockford, IL, USA) and nuclear (anti B23, Thermo-Fisher) extracts. The chemiluminescence detection system (Super Signal^®^ West Pico) was from Thermo-Fisher, Rockford, IL, USA

### 3.10. Statistical Analyses

The results were expressed as the mean ± standard deviation of the mean (SD) when data were combined. For statistical analyses, GraphPad Prism 6.01 software for Windows (GraphPad Software Inc., San Diego, CA, USA) was used: one-way analysis of variance (ANOVA) followed by Bonferroni’s post-hoc test was used for all the experiments. Differences supported by *p* < 0.05 were considered statistically significant.

## 4. Conclusions

In this study, myricetin-3-*O*-α-rhamnoside and 7,8,3′,4′-tetrahydroxy-3,5-dimethoxyflavone (3,5-di-*O*-methyl gossypetin) were isolated and identified from *S. samarangense* leaves. Both compounds show substantial antioxidant activities in vitro. In HaCaT cells, both compounds strongly reduced intracellular ROS accumulation and carbonyl content, and also protected the intercellular GSH levels in keratinocytes after exposure to the toxic agent sodium arsenite (NaAsO_2_). Furthermore, we found that compound **2** exerts its antioxidant activity by stimulating translocation of the transcription factor Nrf-2 to the nucleus. Once in the nucleus, the transcription factor binds to ARE sequences and induces the expression of phase II detoxifying enzymes, i.e., HO-1 and Mn-SOD-3. Finally, we demonstrated that both compounds were able to inhibit IκB-α degradation, thus protecting cells from UVA-induced inflammation. Both compounds may be useful to explore the antioxidant and anti-inflammatory potential of *S. samarangense.*

## Figures and Tables

**Table 1:** ^1^H- (500 MHz) and ^13^C-NMR (125 MHz) data in (MeOD-d_4_) for myricetin-3-*O*-α-rhamnoside (compound **1**) and 7,8,3′,4′-tetrahydroxy-3,5-dimethoxyflavone (compound **2**).

Position	Compound 1		Compound 2	
	δ_H_	δ_C_	δ_H_	δ_C_
2		159.56		155.61
3		136.44		141.29
4		179.80		176.64
5		163.36		154.64
6	6.20 (d, *J* = 2.1 Hz)	100.02	6.45 (s)	97.24
7		166.27		152.93
8	6.36 (d, *J* = 2.1 Hz)	94.88		127.47
8a		158.68		146.94
4a		105.95		108.79
3-OCH3			3.76 (s)	60.36
5-OCH3			3.83 (s)	56.56
1′		122.07		123.42
2′	6.95 (s)	109.70	7.70 (d, *J* = 2.0 Hz)	116.76
3′		147.01		146.39
4′		138.05		149.58
5′		147.01	6.89 (d, *J* = 8.4)	116.42
6′	6.95 (s)	109.70	7.61 (dd, *J* = 8.4 & 2.0 Hz)	122.45
1′′	5.23 (d, *J* = 1.5 Hz)	103.78		
2′′	4.22 (dd, *J* = 1.5 & 3.4 Hz)	72.04		
3′′	3.79 (dd, *J* = 9.5 & 3. 4 Hz)	72.19		
4′′	3.35 (t, *J* = 9.5 Hz)	73.51		
5′′	3.52 (dd, *J* = 9.8 & 6.2 Hz)	72.28		
CH3-rh	0.98 (d, *J* = 6.2 Hz)	17.82		

**Table 2:** Antioxidant activities of myricetin-3-*O*-α-rhamnoside (compound **1**) and 3,5-di-*O*-methyl gossypetin (compound **2**). The EC_50_ values for total extract, ascorbic acid and quercetin are reported for comparison.

	DPPH	FRAP
Sample	(EC_50_ µg/mL)	(mM FeSO_4_ Equivalent/mg Sample)
Compound **1**	3.21	22.9
Compound **2**	3.89	21.08
Total extract [5]	5.80	10
Ascorbic acid	2.94	-
Quercetin	-	23.18

## Abbreviations

ARE, antioxidant response element; AhR, aryl hydrocarbonreceptor; DCF, 2′,7′-dichlorofluorescein; DMEM, Dulbecco’s Modified Eagle’s Medium; DNP, 2,4-dinitrophenol; DNPH, 2,4-dinitrophenylhydrazine; DPPH, 2,2-diphenyl-1-picryl-hydrazyl-hydrate; DTNB, 5,5′-dithiobis-2-nitrobenzoic acid; EDTA, ethylene diamine tetra acetic acid; FoxOs, fork-head box O; FRAP, radical scavenging and ferric reducing antioxidant power; HO-1, heme oxygenase-1; H_2_-DCFDA, 2′,7′-dichlorodihydrofluorescein diacetate; Keap-1, Kelch-like ECH-associated protein 1; Nrf-2, nuclear factor (erythroid-derived 2)-like 2; NQO1, NAD(P)H quinone oxidoreductase1; P-HSP-27, phosphorylated heat shock protein; P-MAPKAPK-2, phosphorylated MAP kinase-activated protein kinase; PPARγ, peroxisome proliferator-activated receptor gamma; P-p38, phosphorylated p38 MAP kinase; ROS, reactive oxygen species; SOD, superoxide dismutase; TBA, thiobarbituric acid; TBARS, TBA reactive substances; TNB, 5-thio-2-nitrobenzoic acid; and γ-GCLC, γ-glutamate-cysteine ligase.
